# Genome Sequences of 11 *Brucella abortus* Isolates from Persistently Infected Italian Regions

**DOI:** 10.1128/genomeA.01402-15

**Published:** 2015-12-17

**Authors:** Giuliano Garofolo, Jeffrey T. Foster, Kevin Drees, Katiuscia Zilli, Ilenia Platone, Massimo Ancora, Cesare Cammà, Fabrizio De Massis, Paolo Calistri, Elisabetta Di Giannatale

**Affiliations:** a National and OIE Reference Laboratory for Brucellosis, Istituto Zooprofilattico Sperimentale dell’Abruzzo e del Molise, Teramo, Italy; b Department of Molecular, Cellular and Biomedical Sciences, University of New Hampshire, Durham, New Hampshire, USA

## Abstract

Bovine brucellosis, typically caused by *Brucella abortus*, has been eradicated from much of the developed world. However, the disease remains prevalent in southern Italy, persisting as a public and livestock health concern. We report here the whole-genome sequences of 11 isolates from cattle (*Bos taurus*) and water buffalo (*Bubalus bubalis*) that are representative of the current genetic diversity of *B. abortus* lineages circulating in Italy.

## GENOME ANNOUNCEMENT

*Brucella abortus* was recognized as the causative agent of bovine brucellosis by Benhard Bang in 1895, distinguishing it as a separate strain from *Brucella melitensis*, the causative agent of Malta fever (1). The number of species in the *Brucella* genus has greatly expanded since then, but *B. abortus* remains among the world’s most important livestock pathogens (2). In the past 40 years, concerted eradication efforts have reduced brucellosis prevalence in cattle in several European countries, yet the disease remains endemic in some Mediterranean countries, causing economic losses for agricultural producers and zoonotic concerns for consumers of contaminated dairy products and animal handlers (3). Despite a countrywide eradication program in Italy, the disease-free status has been achieved only in the central and northern parts of the country. In a previous study, we conducted variable-number tandem-repeat (VNTR) analysis of isolates to understand the genetic relatedness among Italian strains (4). Based on these results and recommended guidelines, we selected a subset of genetically and geographically diverse isolates for whole-genome sequencing (5).

Nine *B. abortus* biovar 3 and two *B. abortus* biovar 1 strains were selected for sequencing, 10 from cattle and one from a water buffalo (strain 8979). Approximately 1 to 5 µg of genomic DNA extracted from each isolate was sheared in a SonicMan microplate sonicator (Brooks Automation, Chelmsford, MA, USA) to produce fragments averaging 600 bp in length. Libraries for Illumina sequencing were prepared with the Kapa high-throughput library preparation kit “with bead” (Kapa Biosystems, Wilmington, MA). The libraries were labeled with 8-bp indices for multiplex sequencing (6). Library quantification was conducted with the Kapa library quantification kit on an ABI Prism 9600 real-time PCR system (Life Technologies, Grand Island, NY). The fragment size distribution was also confirmed with an Agilent DNA high-sensitivity kit for the 2100 Bioanalyzer (Agilent Technologies, Santa Clara, CA, USA). An Illumina MiSeq was used to sequence the libraries, producing 250-bp paired-end reads. The reads were assembled *de novo* with SPAdes 3.0.0 (7) and improved with Pilon 1.8 (8) and SSPACE 3.0 (9). Single-nucleotide polymorphisms (SNPs) were detected relative to the genome of reference strain *B. abortus* 2308 (GenBank accession numbers NC_007618.1 and NC_007624.1) using MUMmer 3.23 (10). The genomes were annotated by NCBI with PGAP 2.10.

The genome assemblies covered an average of 99.61% of the reference genome, with a mean of 16 contigs among assemblies and an *N*_50_ of 434,000 bp. The assemblies averaged 3,161 genes (3,062 coding sequences [CDSs]), representing >99% of the genes in the reference sequence. Further genome annotation results are available in Table 1. For variable sites, 2,116 SNP positions were orthologous to the reference and all of the samples from Italy. In conclusion, our data set will hopefully form the basis of new studies of *B. abortus* in Italy. As a result, whole-genome sequencing is providing new insights into the phylogeography and molecular evolution of Italian isolates of *Brucella*.

**TABLE 1:** Genome annotation statistics

Sample ID	No. of genes	No. of CDSs^a^	No. of pseudogenes	No. of rRNAs^b^	No. of tRNAs	No. of noncoding RNAs	No. of frameshifted genes	No. of SNPs	Accession no.
*Brucella abortus* 2308	3,185	3,084	33	3,3,3	55	4	20		
1046	3,146	3,055	37	1,1,1	50	1	27	1,449	LITY00000000
11796	3,144	3,046	44	1,1,1	50	1	32	1,568	LITX00000000
12183	3,157	3,060	38	4,1,1	52	1	29	1,622	LITW00000000
1365.1	3,160	3,057	40	4,2,4	52	1	30	1,629	LITV00000000
15074.1	3,139	3,042	42	2,1,1	50	1	30	1,615	LJDE00000000
3272	3,164	3,079	30	1,1,1	51	1	22	1,464	LITZ00000000
5586	3,141	3,050	36	1,1,1	51	1	28	1,606	LIUA00000000
8486	3,196	3,072	40	6,6,6	65	1	28	1,442	LIUB00000000
8979.3^c^	3,164	3,079	30	1,1,1	51	1	22	514	LIUC00000000
9060	3,194	3,073	37	6,6,6	65	1	28	1,635	LIUD00000000
9261^c^	3,168	3,073	34	2,5,1	52	1	24	497	LIUE00000000
Mean among assemblies	3,161	3,062	37		54	1	27	1,367	NA^d^

a CDSs, coding sequences.

b Number of 5S, 16S, 23S rRNA genes.

c *B. abortus* biovar 1.

d Not applicable.

### Nucleotide sequence accession numbers.

The draft genome sequences have been deposited in GenBank under the accession numbers in Table 1.
